# An Ankle Joint Flexion and Extension Movement-Monitoring Device Based on Pressure Sensors

**DOI:** 10.3390/mi14122141

**Published:** 2023-11-22

**Authors:** Chunying Xu, Yu Zhou, Jian Ji, Chuliang Wei

**Affiliations:** 1 School of Engineering, Shantou University, Shantou 515063, China; chunyingxu@stu.edu.cn (C.X.); 22yzhou2@stu.edu.cn (Y.Z.); 2 Zhejiang Provincial Institute of Marine Development and Research, Zhoushan 316100, China; jijianzs@sina.com

**Keywords:** ankle pump movement, ankle joint flexion and extension movement monitoring, pressure sensor, STM32

## Abstract

Ankle joint flexion and extension movements play an important role in the rehabilitation training of patients who have been injured or bedridden for a long time before and after surgery. Accurately guiding patients to perform ankle flexion and extension movements can significantly reduce deep vein thromboembolism. Currently, most ankle rehabilitation devices focus on assisting patients with ankle flexion and extension movements, and there is a lack of devices for effectively monitoring these movements. In this study, we designed an ankle joint flexion and extension movement-monitoring device based on a pressure sensor. It was composed of an STM32 microcontroller, a pressure sensor, an HX711A/D conversion chip, and an ESP8266 WiFi communication module. The value of the force and the effective number of ankle joint flexion and extension movements were obtained. An experimental device was designed to verify the accuracy of the system. The maximum average error was 0.068 N; the maximum average relative error was 1.7%; the maximum mean-squared error was 0.00464 N. The results indicated that the monitoring device had a high accuracy and could effectively monitor the force of ankle flexion and extension movements, ultimately ensuring that the patient could effectively monitor and grasp the active ankle pump movement.

## 1. Introduction

**Figure 1:**
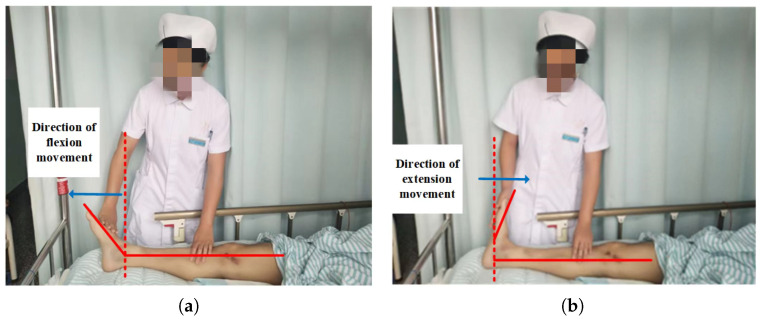
Schematic diagram of ankle joint flexion and extension movement. (**a**) flexion movement; (**b**) extension movement.

Many injuries, and the prolonged bed rest before and after surgery, can easily lead to lower limb deep vein thromboembolism, which greatly affects a patient’s life and can even result in death. Ankle joint flexion and extension movements can effectively avoid this situation. Ankle joint flexion and extension movements are effective measures for preventing venous thromboembolism [1], and they refer to the flexion and extension movements of the ankle joint [2]. A schematic of ankle joint flexion and extension movements is shown in Figure 1. First, the patient lies flat on a bed, exerts maximum force on his/her toes (flexion movement), moves his/her toes towards the outside of his/her body, and maintains this position for 10 s; then, he/she uses force to hook his/her feet up (back extension movement) and move his/her toes towards the inside of his/her body, holding this position for another 10 s. These steps are then repeated continuously for 10 min. The above steps are the complete process for patients performing ankle flexion and extension movements.

Traditional ankle flexion and extension movements generally require medical staff to assist patients in completing them; however, this method has a few drawbacks, such as one medical staff member can only perform ankle flexion and extension movements for one patient at a time, which results in lower efficiency. When patients perform ankle flexion and extension movements, there is a distinction between comfort and maximal strength. Different populations and people in different rehabilitation stages have different maximum strengths. Therefore, it is necessary to monitor the effectiveness of the ankle flexion and extension movements to help patients better complete the ankle flexion and extension movements.

Currently, the research on ankle flexion and extension mainly focuses on ankle pump rehabilitation devices; there is relatively little research on ankle pump movement monitoring. G. Westrich et al. used an Acuson 128XP/10 dual-function ultrasound device with a 5 MHz linear array probe to measure the rate of the pressure rise and the maximum pressure reached during ankle flexion and extension movements to evaluate the peak venous velocity and venous volume [3]. However, this device can only measure the maximum pressure and cannot display the real-time pressure value during ankle joint flexion and extension movements, and there are also issues such as the high equipment costs. Yoon J. Y. et al. used a goniometer to measure the angles of extension and flexion during ankle flexion and extension movements [4]. Kim et al. compared the ankle joint angles measured using wearable devices with the ankle joint angles obtained using movement-capture systems during running and verified the ability of inertial measurement units (IMUs) to accurately measure ankle joint angles, thereby detecting the effectiveness of ankle joint flexion and extension movements [5]. Knyazev A. A. et al. developed an ankle joint mechanical treatment device based on a parallel robotic arm. The measuring instrument built into the device platform monitors the interaction force between the foot and the platform at three points. The movement control unit of the mobile platform generates a control voltage based on the reference model, corrects stress, and compares the changes in the ankle physiological parameters during rehabilitation to effectively monitor the ankle flexion and extension movements [6].

Research has been conducted on the monitoring of ankle flexion and extension movements. Al-Quraishi et al. used electromyography signals for pattern recognition, which was mainly divided into four stages: signal detection and preprocessing, feature extraction, dimensionality reduction, and classification. They used three classifiers (linear discriminant analysis, K-nearest neighbors, and multilayer perceptron) to classify four types of ankle joint movements [7]. Hideki Toda et al. used a device that can achieve affected ankle joint stretching by using the angle of the healthy ankle joint as a trigger to move the foot plate connected to a linear actuator. They used two acceleration sensors to measure the ankle joint rotation angle on the affected and healthy sides. Compared to the previously used button control, healthy side control can achieve a smooth and stable process of pressing the affected side’s sole. The proposed system does not require a physical therapist during the treatment process, making it possible for the ankle joint to self-recover [8]. Zhetenbayev et al. provided a portable handheld device for ankle movement training during rehabilitation [9]. This portable device is based on the extensive movement of the human ankle provided in rehabilitation. A numerical model was used to study the motion amplitude of the ankle joint as a specific motion feature in order to monitor the flexion and extension of the ankle joint. However, there are currently no devices that monitor forces during flexion and extension on the market and in research.

Currently, pressure sensors are widely used in human motion monitoring. Zhang et al. assembled and designed a pressure sensor composed of micro-convex polydimethylsiloxane, MXene nanosheets/Ag nanoflower films, and flexible interdigital electrodes to monitor the wearer’s movement status and physiological signals [10]. They developed an intelligent glove through a straightforward method, integrating it with a 3D model for wireless and precise detection of hand gestures. However, this type of pressure sensor tends to be expensive, making it less suitable for widespread applications. Yang et al. designed a shoe inner sensor pad attached to the shoe lining based on a friction electric nanogenerator, which was used to monitor the stress distribution on the foot in real-time and monitor the pressure generated by humans during movement [11]. Yu et al. designed a piezo-triboelectric pressure sensor based on a triboelectric nanogenerator and piezoelectric nanogenerator for real-time monitoring of human joint bending motion [12]. Lee et al. developed nanomesh pressure sensors capable of monitoring finger pressure without detectable effects on human sensation [13]. Wu et al. designed a low-cost flexible pressure sensor with a positive resistance–pressure response based on laser scribing graphene, enabling the detection of various physiological signals and human movements [14]. Gerlach et al. selected a multiwalled carbon nanotube–polydimethylsiloxane composite material as the pressure sensorto monitor changes in plantar pressure during human movements [15]. Xiong et al. developed a flexible and highly sensitive capacitive pressure sensor, making significant progress in monitoring physiological signals and robot gripper movements [16]. Liu et al. used a large-area all-textile-based pressure sensor to monitor finger movements and recognize gestures [17]. Yang et al. applied a flexible microfluidic pressure sensor based on the frictional charge at the liquid–solid interface during liquid flow in microfluidic channels to monitor finger movement [18].

The force value is an important standard for evaluating the effectiveness of the ankle joint flexion and extension movements. However, there is a shortage of pertinent monitoring research and products. Moreover, there is currently a mismatch between the demand for monitoring devices for ankle joint flexion and extension movements and the medical resources available for a large number of long-term bedridden patients. There is an urgent need to design a low-cost and easy-to-monitor ankle joint flexion and extension movement-monitoring device to assist long-term bedridden patients in rehabilitation training. Pressure sensors have not been utilized for monitoring ankle joint flexion and extension movements. Therefore, this paper proposes an active ankle joint flexion and extension movement-monitoring device based on pressure sensors. The monitoring system offers the following advantages: (1) It accurately monitors force values during the ankle joint flexion and extension movements process, assisting users and medical staff in observing and evaluating movements and rehabilitation status and facilitating timely adjustments for improved recovery. (2) The monitoring device is cost-effective, significantly reducing the manufacturing costs.

The remainder of this paper is organized as follows. Section 2 introduces the implementation method for monitoring the force values during ankle flexion and extension movements based on a pressure sensor. Section 3 presents the experiment and accuracy analysis. Finally, Section 4 concludes the paper.

## 2. Method

**Figure 2:**
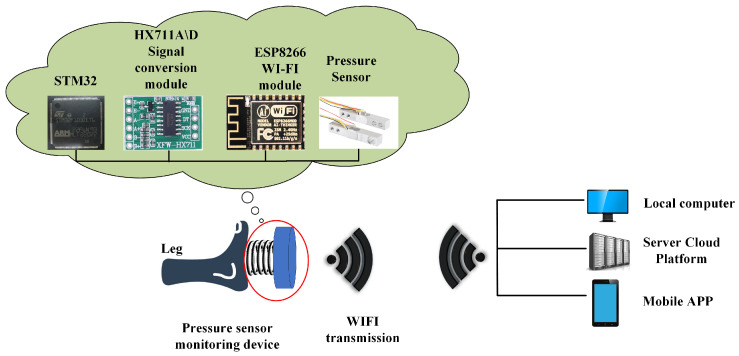
Structure of an active ankle flexion and extension movement system based on pressure sensors.

The system’s composition is shown in Figure 2 and includes an STM32 microcontroller (STMicroelectronics, Paris, France), a pressure sensor [19], an HX711 A/D signal conversion module (AVIA Semiconductor, Shenzhen, China) [20], an ESP8266 module (Guangzhou Xingyi Electronic Technology Co., Ltd., Guangzhou, China) [21], and an upper computer module.

**Figure 3:**
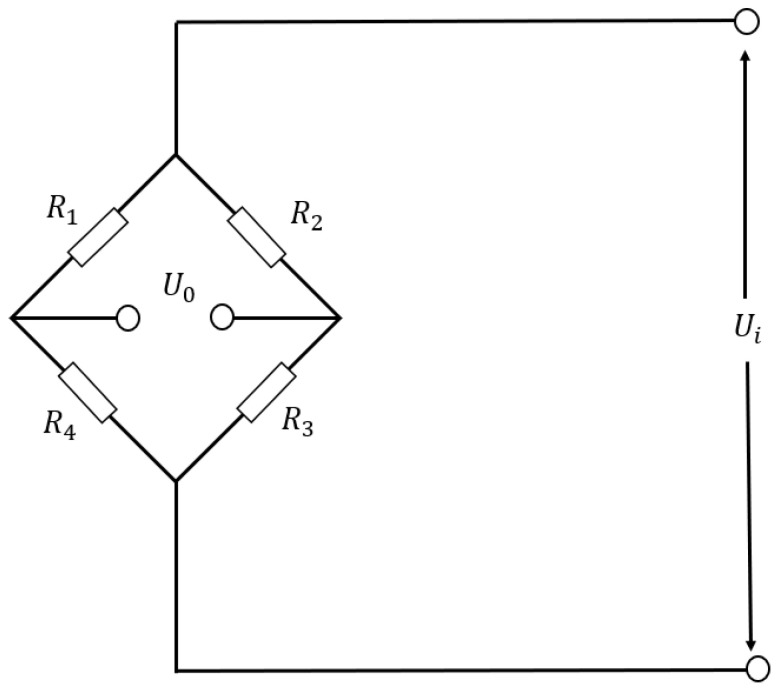
Internal circuit diagram of pressure sensor.

The STM32 microcontroller was the STM32F103ZET6, renowned for its rich peripheral interfaces, low-power design, and security features. The ESP8266 module is an ultra-low-power module specifically designed for serial WiFi communication, boasting characteristics such as low power consumption, high stability, and excellent integration. The HX711 A/D signal conversion module’s function is to convert the pressure signal from the pressure sensor. It offers advantages such as high integration, rapid response speed, and robust anti-interference capabilities. The pressure sensor employed was a resistance strain sensor, leveraging the resistance strain effect to induce changes in the conductor’s resistance with mechanical deformation. It possesses characteristics such as a wide measurement range, high resolution and sensitivity, high accuracy, and strong anti-interference capabilities, making it well-suited for ankle flexion and extension movement monitoring and efficient force value detection. The selected pressure sensor in this article has an accuracy of 0.01 N. The circuit diagram of the pressure sensor is depicted in Figure 3.

As shown in Figure 3, the pressure sensor primarily consists of a Wheatstone bridge. When the output end of the bridge is connected to an infinite load resistance, the output end can be considered as an open circuit. In this scenario, the DC bridge is referred to as a voltage bridge, exclusively providing voltage output. By disregarding the internal resistance of the power supply, Formula (1) can be obtained from the principle of voltage division:

(1) $$\begin{array}{cc} U_{o} & =U_{i}\left(\frac{R_{1}}{R_{1}+R_{4}}-\frac{R_{2}}{R_{2}+R_{3}}\right)\\ \; & =U_{i}\left(\frac{R-\Delta R}{R-\Delta R+R+\Delta R}-\frac{R+\Delta R}{R+\Delta R+R-\Delta R}\right)\\ \; & =U_{i}\left(\frac{R-\Delta R}{R-\Delta R+R+\Delta R}-\frac{R+\Delta R}{R+\Delta R+R-\Delta R}\right)\\ \; & =\varepsilon U_{i} \end{array}$$

In Formula (1), $U_{o}$ represents the output voltage, $U_{i}$ represents the input voltage, *R* represents the resistance value of the resistance, ΔR represents the change in the resistance value of the resistance strain gauge when the pressure sensor is under force, and ε represents the sensitivity coefficient of the resistance strain gauge.

To ensure that force values during ankle flexion and extension movements, particularly during extension movements, can be accurately applied to the pressure sensor, the monitoring device was securely affixed to a wall. A foot was fastened to a non-elastic strap, which, in turn, was linked to a spring. The other end of the spring was anchored to the pressure sensor device. As a patient executed ankle flexion and extension movements, the ankle compressed or stretched the spring, thereby compressing or pulling the pressure sensor.

**Figure 4:**
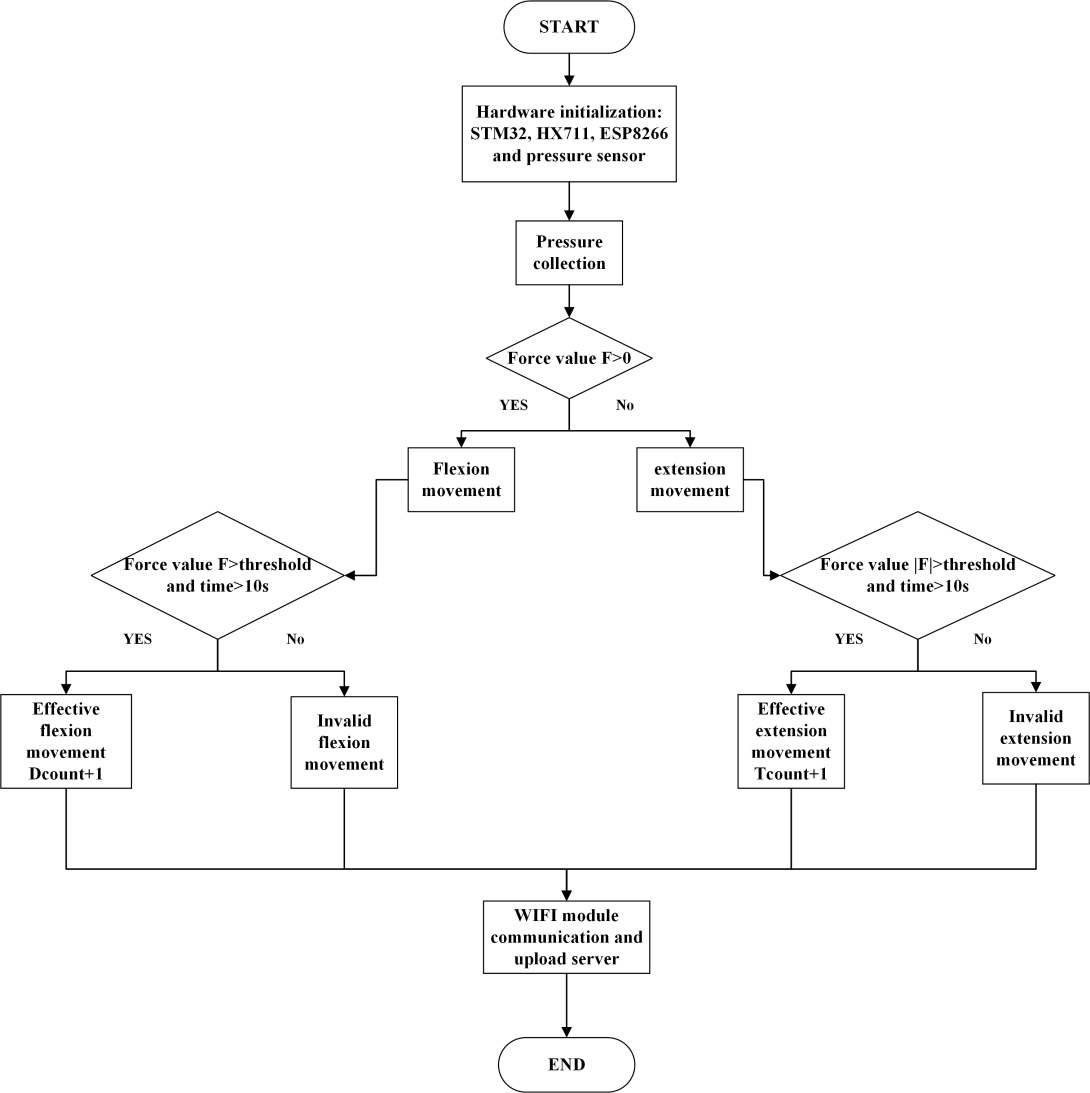
Flow chart of ankle joint flexion and extension movement-monitoring system based on pressure sensor.

The workflow diagram of the system is shown in Figure 4. Initially, the hardware components, comprising a microcontroller, an HX711 A/D conversion module, an ESP8266 WiFi communication module, and a pressure sensor, were initialized. Upon receiving the start command via the WiFi module, the STM32 microcontroller triggers the HX711 module to gather electrical information produced by the pressure sensor. Simultaneously, the A/D signal-conversion module collects the force value of the ankle joint flexion and extension movements. The microcontroller then processes the pressure values corresponding to the patient’s flexion movement, the pulling force during extension movement, and the effective number of movements. Finally, the microcontroller utilizes the WiFi communication module to upload the collected data to the server. The mobile app accesses these data from the server’s cloud platform, enabling real-time monitoring of ankle joint flexion and extension movements. Both users and medical staff can monitor and evaluate real-time and historical data through the server or mobile app, facilitating timely adjustments for enhanced recovery.

**Figure 5:**
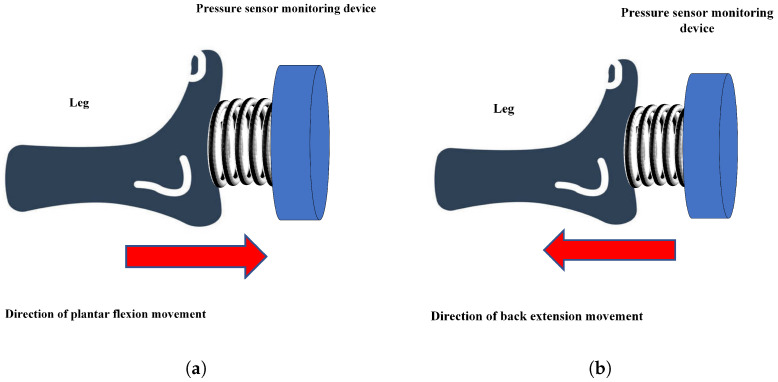
Schematic diagram of ankle joint flexion and extension movement. (**a**) flexion movement; (**b**) extension movement.

As shown in Figure 5, the directions of flexion and extension movements are exactly opposite; thus, there are positive and negative values for the movement force. A positive force value represents the direction of flexion movement, and a negative force value represents ankle joint extension movement. We set the effective number of flexion movements as Dcount and the effective number of extension movements as Tcount. The maximum force value for each user is not the same, and the user can first record the absolute value of his/her maximum force value (because the force value is positive or negative) as a threshold. In subsequent movements, when the absolute value of the force value is greater than the threshold and the duration exceeds 10 s, the plantar flexion or back extension movement is considered effective, and the corresponding number of effective times Dcount is increased by 1. In contrast, if the absolute value of the movement force is less than the set threshold or the duration of the movement does not meet the conditions, the value of Dcount remains unchanged.

## 3. Experiment and Accuracy Analysis

### 3.1. Analysis of Extension Movement Accuracy

**Figure 6:**
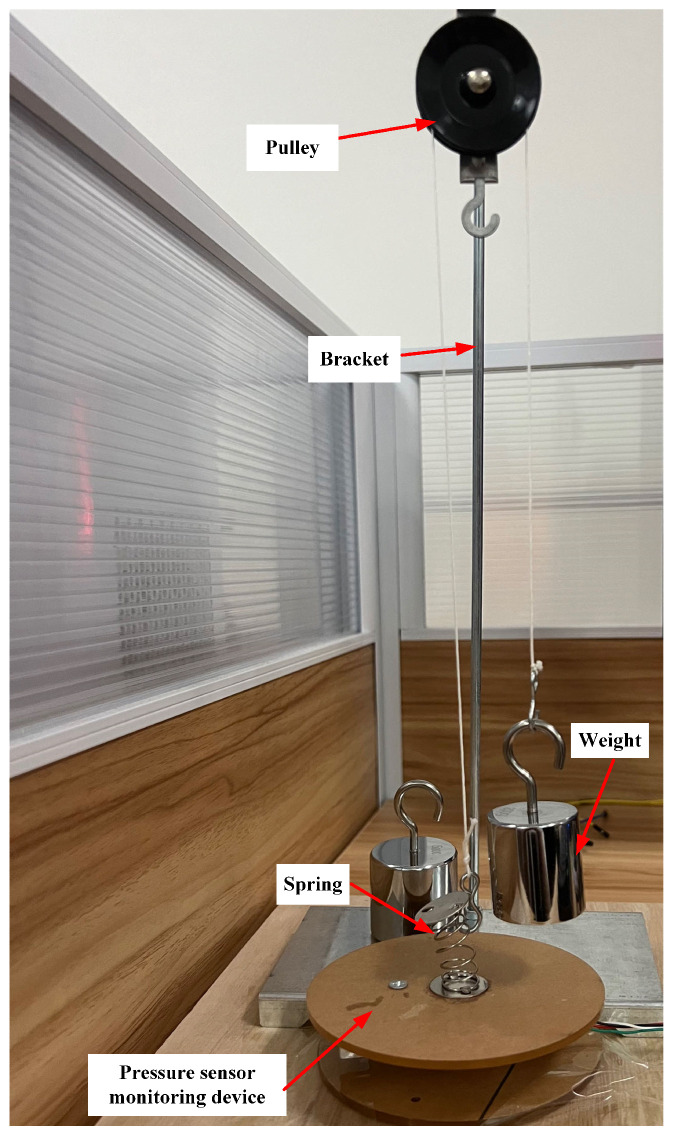
Experimental device diagram for analyzing the monitoring accuracy of extension force value.

The extension movement involves hooking the foot upward with force, and the pressure sensor is in a pulled state. To verify the accuracy of monitoring the back extension movement, the force value of the back extension movement was simulated using a pulley device. As shown in Figure 6, it included a bracket, fixed pulley, weight, string, and ankle joint flexion and extension movement-monitoring device. The pulley was fixed to a bracket. One end of the thin rope (without elasticity) was connected to a weight, and the other end was connected to the monitoring device. Simultaneously, the device was fixed to a desktop. To simulate the different force values of back extension movements, five weights with different masses were used to complete the experiment. The weights were 100 g (1 N), 200 g (2 N), 300 g (3 N), 400 g (4 N), and 500 g (5 N), respectively. Because of the influence of its own weight, the weight will pull the string to move, thereby pulling the spring and generating a pull force. Based on relevant physical knowledge, it can be seen that the pull force applied to the monitoring device was equal to the mass of the weight. Ten experiments were performed for each weight, and the relevant data were recorded. The weight mass and force values of the monitoring device were used to calculate the average and mean-squared errors, respectively. The results are summarized in Table 1.

From Table 1, it can be seen that the monitoring accuracy of the extension movement was high, with an average error of 0.023 N at the maximum and 0.001 N at the minimum. The maximum and minimum average relative errors were 0.9% and 0.1%, respectively. The maximum and minimum mean-squared errors were 7.5 × $10^{-4}$ N and 1 × $10^{-5}$ N, respectively. It could effectively monitor the force of the ankle joint extension movement.

### 3.2. Analysis of Flexion Movement Accuracy

**Figure 7:**
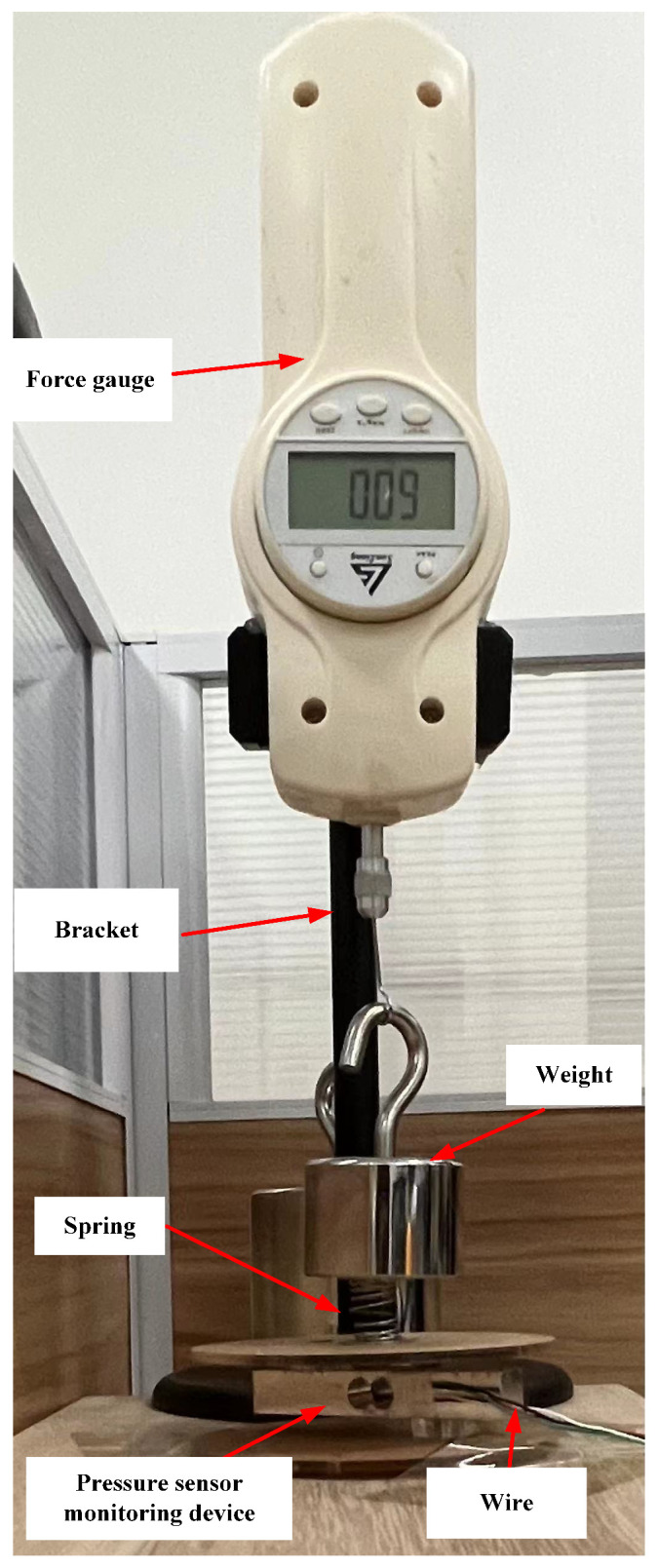
Experimental device diagram for analyzing the monitoring accuracy of flexion force value.

The flexion movement occurs when force is applied to press down the toes, causing the pressure sensor to be in a compressed state. Figure 7 illustrates the accuracy analysis of the experimental setup, encompassing the bracket, a force gauge, weights, and the monitoring device. The monitoring device was securely affixed to a desktop. However, when the weight was directly pressed onto the spring, it tended to slip off. Consequently, the weight was suspended from a force gauge attached to a bracket. The application of pressure force to the spring and monitoring device was achieved by adjusting both the weight and the force gauge, as depicted in Formula (2).

(2) $$F=mg-F_{0}$$

In Formula (2), *m* is the mass of the weight itself and *g* is the acceleration due to gravity and has a value 10 m/s${}^{2}$. $F_{0}$ represents the force exerted by the force gauge. In this experiment, five weights with different masses were used: 200 g, 300 g, 400 g, 500 g, and 1 kg. Ten experiments were performed for each weight, and the relevant data were recorded. The ith pressure value $F_{i}$ (*i* = 1, 2, 3..., 10) measured by the monitoring device and the actual pressure *F* were used to calculate the average and mean-squared errors. The results are summarized in Table 2. The range of the force gauge was 10 N; the resolution was 0.01 N; the accuracy was ±0.05 N.

As shown in Table 2, the monitoring accuracy of the flexion movement was high, with average maximum and minimum errors of 0.068 N and 0.002 N, respectively. The maximum and minimum average relative errors were 1.7% and 0.2%,respectively. The maximum and minimum mean-squared errors were 4.64 × $10^{-3}$ N and 2 × $10^{-5}$ N, respectively. It could effectively monitor the force of the ankle joint flexion movement.

In summary, the device developed in this study can accurately monitor the force values of the ankle joint flexion and extension movements.

**Figure 8:**
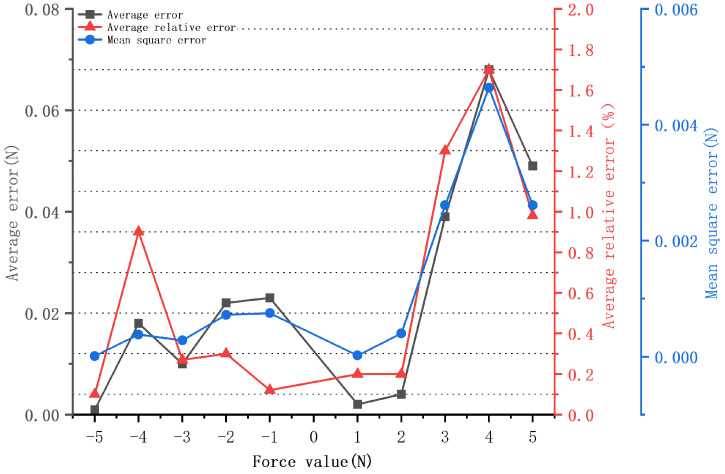
Analysis curve for the accuracy of force values during flexion and extension movements.

The error analysis curve of the flexion and extension movement is shown in Figure 8. The positive values denote flexion movement, while negative values represent extension movement. The black curve signifies the average error; the red curve represents the average relative error; the blue curve indicates the mean-squared error. As shown in Figure 8, for flexion movements, the maximum average error, average relative error, and mean-squared error were 0.068 N, 1.7%, and 4.64 × $10^{-3}$ N, respectively. The minimum average error, average relative error, and mean-squared error were 0.002 N, 0.2%, and 2 × $10^{-5}$ N, respectively. For extension movements, the maximum average error, average relative error, and mean-squared error were 0.023 N, 0.9%, and 7.5 × $10^{-4}$ N, respectively, while the minimum average error, average relative error, and mean-squared error were 0.001 N, 0.1%, and 1 × $10^{-5}$ N, respectively. The results indicated that the monitoring device had high accuracy and could effectively monitor the force of the ankle flexion and extension movements.

### 3.3. Experimental Data Record of Ankle Joint Flexion and Extension Movement

**Figure 9:**
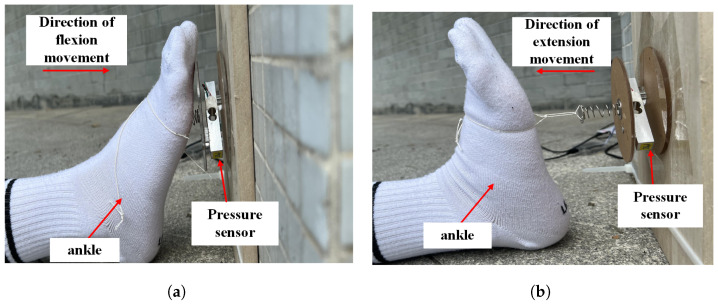
Experimental diagram of ankle joint flexion and extension movement. (**a**) flexion movement; (**b**) extension movement.

As depicted in Figure 9, one side of the spring was affixed to the pressure sensor, while the other side was connected to the foot. Subsequently, the flexion and extension movements of the ankle joint were initiated. In the flexion movement, the sole of the foot moved towards the outside of the body, compressing the spring and generating pressure. Conversely, in the extension movement, the foot moved towards the inside of the body, causing the spring to move in tandem. Since the opposite side of the spring was anchored to the pressure sensor, the spring pulled the upper side of the pressure sensor, resulting in the deformation and the generation of tensile force values. We set the threshold for both flexion and extension movements to 2 N and record the maximum force values and the corresponding effective numbers for 10 flexion and extension movements, respectively, in Table 3.

From the experimental results in Table 3, we can see the maximum force value and effective number of times generated during the flexion and extension movements. Among the 10 effective flexion movements, the maximum effective force value was 6.35 N, while the minimum effective force value was 5.83 N. The average effective force value for these 10 flexion movements was 6.167 N. In the case of the 10 effective extension movements, the maximum effective force value was 5.95 N, and the minimum effective force value was 3.57 N. The average effective force value for these 10 extension movements was 4.039 N. Users can set thresholds based on their individual needs to monitor the ankle joint flexion and extension movements. The device designed in this article can monitor a maximum force value of 50 N and a minimum force value of 0.01 N. The experimental results demonstrated that our proposed ankle joint flexion and extension movement-monitoring device, based on pressure sensors, is effective at monitoring these movements.

## 4. Conclusions

Ankle joint flexion and extension movements are very important for the rehabilitation of bedridden patients. In this study, a monitoring device for ankle joint flexion and extension movements was designed, leveraging a pressure sensor to accurately measure force values during these movements. The maximum and minimum average error values for flexion movement were 0.068 N and 0.0001 N, respectively. The maximum and minimum mean-squared error values were 2.61 × $10^{-3}$ N and 2 × $10^{-5}$ N, respectively. For monitoring extension movement, the average error was 0.023 N and 0.0001 N within different force ranges. The maximum and minimum mean square deviation values were 7.5 × $10^{-4}$ N and 1 × $10^{-5}$ N, respectively. These results indicated that the device satisfied the conditions for effectively monitoring the ankle flexion and extension movements. Furthermore, this device had the capability to count the effective number of ankle joint flexion and extension movements and transmit the data to mobile applications, establishing it as an effective ankle flexion and extension monitoring device. In the future, the monitoring system can be integrated with rehabilitation devices, including the incorporation of load-bearing modules to enhance users’ performance in rehabilitation training. Additionally, an attitude sensor can be incorporated into the monitoring system. This enhancement would enable the simultaneous measurement of both the force and attitude angle during ankle joint flexion and extension movements, thereby achieving a more-comprehensive monitoring capability.

## Figures and Tables

**Table 1:** Experiment data and accuracy analysis of the extension force value.

Weight Mass	First Force Value (N)	Second Force Value (N)	Third Force Value (N)	Fourth Force Value (N)	Fifth Force Value (N)	Sixth Force Value (N)	Seventh Force Value (N)	Eighth Force Value (N)	Ninth Force Value (N)	Tenth Force Value (N)	Average Error (N)	Average Relative Error (%)	Mean-Squared Error (N)
100 g (1 N)	1.01	1.00	1.00	1.00	1.00	1.00	1.00	1.00	1.00	1.00	0.001	0.1	1 × $10^{-5}$
200 g (2 N)	1.97	1.98	1.98	1.98	1.98	1.98	1.98	1.98	1.99	2.00	0.018	0.9	3.8 × $10^{-4}$
300 g (3 N)	3.03	3.04	3.01	3.00	3.01	3.00	3.00	3.00	3.00	2.99	0.01	0.27	2.8 × $10^{-4}$
400 g (4 N)	3.95	4.00	4.02	4.03	4.00	3.98	3.99	3.98	3.96	3.97	0.022	0.3	7.2 × $10^{-4}$
500 g (5 N)	4.97	4.96	5.01	4.99	5.02	4.97	5.00	4.97	5.00	5.05	0.023	0.12	7.5 × $10^{-4}$

**Table 2:** The experiment data and accuracy analysis of the flexion force value.

Weight Mass	Force Value $F_{0}$ (N)	Actual Pressure *F* (N)	Pressure Value $F_{1}$ (N)	Pressure Value $F_{2}$ (N)	Pressure Value $F_{3}$ (N)	Pressure Value $F_{4}$ (N)	Pressure Value $F_{5}$ (N)	Pressure Value $F_{6}$ (N)	Pressure Value $F_{7}$ (N)	Pressure Value $F_{8}$ (N)	Pressure Value $F_{9}$ (N)	Pressure Value $F_{10}$ (N)	Average Error (N)	Average Relative Error (%)	Mean Square Error (N)
200 g (2 N)	1.00	1.00	1.00	1.00	1.00	1.00	1.00	1.01	1.01	1.00	1.00	1.00	0.002	0.2	2 × $10^{-5}$
300 g (3 N)	1.00	2.00	1.99	1.99	1.99	1.99	2.00	2.00	2.00	2.00	2.00	2.00	0.004	0.2	4 × $10^{-4}$
400 g (4 N)	1.00	3.00	3.01	3.08	3.11	3.06	3.04	3.03	3.03	3.02	3.01	3.00	0.039	1.3	2.61 × $10^{-3}$
500 g (5 N)	1.00	4.00	4.07	4.07	4.07	4.07	4.07	4.06	4.07	4.07	4.07	4.07	0.068	1.7	4.64 × $10^{-3}$
1 kg (10 N)	5.00	5.00	5.04	5.05	5.02	5.06	5.08	5.05	5.05	5.05	5.04	5.05	0.049	0.98	2.61 × $10^{-3}$

**Table 3:** Experimental data on ankle joint flexion and extension movements.

Number of Experiments	The Maximum Force Value of Flexion Movement (N)	The Maximum Force Value of Extension Movement (N)	Effective Number of Flexion Movements (Dcount)	Effective Number of Extension Movements (Tcount)
First	6.05	3.57	1	1
Second	5.83	3.65	2	2
Third	6.35	3.61	3	3
Fourth	6.31	3.78	4	4
Fifth	6.24	4.12	5	5
Sixth	6.13	4.03	6	6
Seventh	6.25	3.84	7	7
Eighth	6.33	3.89	8	8
Ninth	6.23	5.95	9	9
Tenth	5.95	3.95	10	10

## Data Availability

The data presented in this study are available upon request from the corresponding author.
